# *QuickStats:* Percentage of Children Aged <18 Years with a Food or Digestive Allergy in the Past 12 Months,* by Age Group — National Health Interview Survey, 2007–2018^†^

**DOI:** 10.15585/mmwr.mm6838a6

**Published:** 2019-09-27

**Figure:**
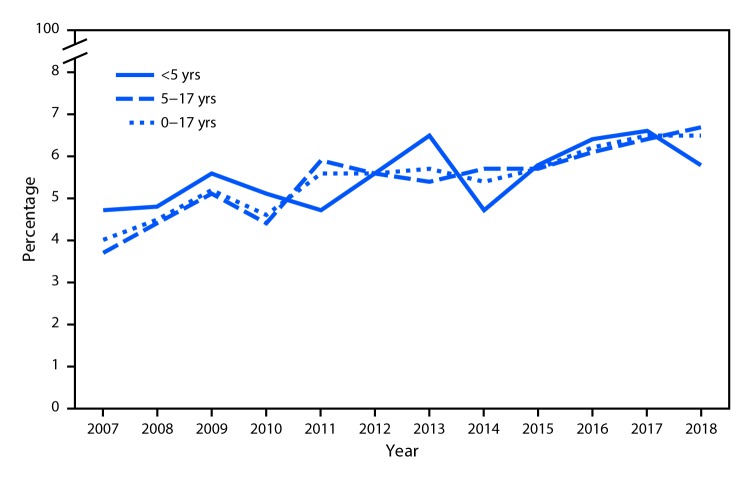
During 2007 to 2018, the percentage of children aged 0–17 years with a food or digestive allergy in the past 12 months increased from 4.0% in 2007 to 6.5% in 2018. Among children aged <5 years, the percentage of food or digestive allergies increased from 4.7% to 5.8%, and among children aged 5–17 years, the percentage of food or digestive allergies also increased from 3.7% to 6.7%.

**Source:** National Health Interview Survey, 2007–2018 data. https://www.cdc.gov/nchs/nhis.htm.

